# Gypsophile Chemistry Unveiled: Fourier Transform Infrared (FTIR) Spectroscopy Provides New Insight into Plant Adaptations to Gypsum Soils

**DOI:** 10.1371/journal.pone.0107285

**Published:** 2014-09-15

**Authors:** Sara Palacio, Matt Aitkenhead, Adrián Escudero, Gabriel Montserrat-Martí, Melchor Maestro, A. H. Jean Robertson

**Affiliations:** 1 Instituto Pirenaico de Ecología (IPE-CSIC), Jaca, Huesca, Spain; 2 The James Hutton Institute, Aberdeen, Scotland, United Kingdom; 3 Biodiversity and Conservation Group, E.S.C.E.T., Universidad Rey Juan Carlos, Móstoles, Madrid, Spain; 4 Instituto Pirenaico de Ecología (IPE-CSIC), Zaragoza, Spain; University of Quebect at Trois-Rivieres, Canada

## Abstract

Gypsum soils are among the most restrictive and widespread substrates for plant life. Plants living on gypsum are classified as gypsophiles (exclusive to gypsum) and gypsovags (non-exclusive to gypsum). The former have been separated into wide and narrow gypsophiles, each with a putative different ecological strategy. Mechanisms displayed by gypsum plants to compete and survive on gypsum are still not fully understood. The aim of this study was to compare the main chemical groups in the leaves of plants with different specificity to gypsum soils and to explore the ability of Fourier transform infrared (FTIR) spectra analyzed with neural network (NN) modelling to discriminate groups of gypsum plants. Leaf samples of 14 species with different specificity to gypsum soils were analysed with FTIR spectroscopy coupled to neural network (NN) modelling. Spectral data were further related to the N, C, S, P, K, Na, Ca, Mg and ash concentrations of samples. The FTIR spectra of the three groups analyzed showed distinct features that enabled their discrimination through NN models. Wide gypsophiles stood out for the strong presence of inorganic compounds in their leaves, particularly gypsum and, in some species, also calcium oxalate crystals. The spectra of gypsovags had less inorganic chemical species, while those of narrow gypsum endemisms had low inorganics but shared with wide gypsophiles the presence of oxalate. Gypsum and calcium oxalate crystals seem to be widespread amongst gypsum specialist plants, possibly as a way to tolerate excess Ca and sulphate. However, other mechanisms such as the accumulation of sulphates in organic molecules are also compatible with plant specialization to gypsum. While gypsovags seem to be stress tolerant plants that tightly regulate the uptake of S and Ca, the ability of narrow gypsum endemisms to accumulate excess Ca as oxalate may indicate their incipient specialization to gypsum.

## Introduction

The ability of plants to survive in substrates with limiting conditions for plant life has intrigued biologists since early times [1], [2]. Gypsum soils are amongst the most widespread special substrates, extending over 100 million hectares [3], [4], but yet they have received comparatively less attention than other substrates such as serpentines, saline or calcicolous soils [5]. Gypsum soils develop from gypsic rocks in arid and semi-arid areas where low precipitation prevents gypsum from being leached [6]. Together with the arid conditions, gypsum soils have particularly stressful physical and chemical properties for plant life including the presence of hard soil crusts, high mechanical instability, low soil porosity, extreme nutritional deficits, high concentration of sulphates and moderate salinity [7], [8], [9], [10]. As a consequence, they are among the most restrictive soils for plants [6]. Nevertheless, the adverse conditions of gypsum soils contrast with the rich and specialized flora they shelter, comprising diverse arrays of narrow endemic and rare plants in arid and semiarid regions, many of which are threatened or endangered and constitute a global conservation biodiversity concern [6], [11].

Depending on their specificity to gypsum soils, plants can be classified as gypsophiles, i.e. plants growing exclusively on gypsum substrates, or gypsovags, i.e. plants growing both in and out of gypsum [11]. Despite the recent efforts devoted to understand plant life on gypsum, the mechanisms displayed by plants to become competitive on gypsum soils are still not fully understood [5]. Early investigations showed that the chemical composition of gypsophiles and gypsovags differs, with the former showing higher concentration of certain nutrients (N, P, Ca, S) and total ashes than the latter [12], [13], [14]. Previous studies also showed that gypsophile seedlings (like *H. squamatum* and *L. subulatum*) show a higher ability than gypsovags to surpass the physical soil crust characteristic of gypsum soils, and hence are better adapted to cope with the physical limitations of gypsum soils [15]. More recently, however, it was found that gypsophiles could be segregated in two different groups of plants, with distinct chemical composition and different putative ecological strategies [16]. These authors found differences in the composition of narrowly and widely distributed gypsophiles, the former being more similar to the chemical composition of gypsovags. According to Gankin and Major's interpretation of edaphism origin [17], narrowly distributed gypsophiles (similarly to gypsovags) would fit the *refuge* model, being stress tolerant species not specifically adapted to gypsum soils that avoid competition in marginal soils, while widely distributed gypsophiles would fit the *specialist* model, being specifically adapted to gypsum and dramatically lowering their performance in other soils [16]. The increased concentration of Ca and S of widely distributed gypsophiles suggests the existence of certain (still unexplored) physiological adaptations to cope with the excess of calcium sulphate in gypsum soils [13], [16]. The high concentrations of certain nutrients (such as N or P) of wide gypsophiles are also intriguing, as gypsum soils are inherently nutrient poor [18], [19].

The results of the study by Palacio *et al.* [16] are crucial since they show that various chemical strategies are feasible for plants living exclusively on gypsum soils. However, they are solely based on the elemental composition of plant species. To know if the generality of their conclusions stands with more comprehensive analyses of plant biochemistry, biochemical fingerprinting techniques allowing for the identification of the functional chemical groups of plants are needed. Such approaches could also shed light on the biochemical and physiological adaptations of different groups of plants to survive on gypsum soils. For example, they could inform on the biochemical mechanisms of wide gypsophiles to accumulate S, Ca, N or P. Such information is critical to understand plant life on gypsum substrates.

Fourier transform infrared (FTIR) spectroscopy is a powerful tool for the chemical analysis of biological samples [20]. This technique offers a fast, cost-effective, non-destructive way of obtaining a biochemical fingerprint of samples, where the main functional groups and bonds can be identified, thus giving structural information on the chemical compounds present rather than just elemental information. Crucially, both organic and inorganic compounds have features in the FTIR spectrum, allowing not only identification of the main organic constituents of plant material but also characterisation of the forms of inorganic compounds present in the plants [21]. In addition, by comparing the replication of FTIR spectra for different plants of the same species grown in the same conditions and localities, the technique provides a means of assessing the variability of the chemical profile among individuals.

FTIR spectroscopy is a widely used tool in plant biological studies [20]. It has been used to detect changes in plant chemistry in relation to fertilization [22], [23], [24] or heavy metal addition [25], [26], [27]. FTIR spectra analysed through artificial neural networks (NN) show a high potential to discriminate biological samples. For instance, [28], [29] and [30] applied NN to FTIR spectral data in order to distinguish between plant species, using a range of different kinds of source material. Despite the huge potential and increasing use of FTIR in plant studies, it has never been applied to the study of edaphic endemisms before.

The aim of this study was to compare the identity of the main chemical groups present in the leaves of plants with different specificity to gypsum soils and to explore the ability of FTIR spectra analyzed with NN to discriminate groups of gypsum plants. We hypothesize that: 1) data obtained by FTIR spectroscopy will show a high correlation with the elemental composition of plants. Consequently, owing to the observed differences in the mineral composition of gypsum plants in previous studies, we further hypothesize that 2) narrow gypsum endemisms will show a similar FTIR spectral composition to gypsovags, while widely distributed gypsophiles will show distinct spectral features.

## Materials and Methods

### Species and study sites

Species and study sites generally followed [16], except for the addition of the gypsovag *Thymelaea tinctoria*, and included fourteen species for analysis: five gypsovags and nine gypsophiles. The latter comprised most woody gypsophile species of study areas, including five widely distributed gypsophiles and four narrow gypsum endemics (Table 1). The distinction between both types of gypsophiles was made according to the extent of their distribution area: widely distributed gypsophiles were species that showed a wide distribution range in the Iberian Peninsula, comprising almost every gypsum outcrop in this large territory, whereas narrow gypsum endemics were species that showed a limited number of populations, growing only in one of the gypsum areas of the Iberian Peninsula. In order to minimize bias due to variations in plant functional strategies, all study species were shrubs or sub-shrubs, which are prevalent growth forms in gypsum outcrops [6], [31], and had a similar branch morphology and architecture (for further details see [16]). None of the species included in this study is endangered or protected [32].

**Table 1:** Location and characteristics of study species and sites.

Species	Species code	Species type^a^	N^b^	Sampling sites^c^	UTM	Altitude (m a.s.l.)	Date of sampling	Subst.^d^
*Centaurea hyssopifolia* Vahl.	Ch	NE	4	Chinchón (M)	VK6247	640	15-Mar-10	G
*Gypsophila struthium* L. subsp. *hispanica* (Wilk.) G. López	Gh	G	5	Villamayor (Z)	XM8820	300	19-Feb-10	G
*Helianthemum marifolium* (L.) Mill. subsp. *conquense* Borja and Rivas Goday	Hco	NE	7	Tendilla (Cu)	WK4214	780	3-Mar-03	G
*Helianthemum squamatum* (L.) Pers.	Hsq	G	5	Villamayor (Z)	XM8718	290	21-Jan-03	G
*Herniaria fruticosa* L.	Hf	G	C	Villamayor (Z)	XM8820	300	20-Jan-03	G
							19-Feb-10	
					XM8718	290	21-Jan-03	G
*Lepidium subulatum* L.	Lsb	G	5	Villamayor (Z)	XM8820	320	13-Jan-03	G
							19-Feb-10	
*Linum suffruticosum* L.	Lsf	GV	5	Villamayor (Z)	XM8820	320	7-Jan-03	G
*Ononis tridentata* L.	Ot	G	5	Villamayor (Z)	XM8820	300	20-Jan-03	G
							19-Feb-10	
*Rosmarinus officinalis* L.	Ro	GV	5	Peñaflor (Z)	XM8626	300	24-Jan-03	G
*Salvia lavandulifolia* Vahl.	Sl	GV	5	Villamayor (Z)	XM8820	320	21-Jan-03	G
*Teucrium polium* L. subsp. *capitatum* (L.) Arcangelli	Tc	GV	5	Chinchón (M)	VK6247	640	6-Feb-03	G
*Teucrium pumilum* L.	Tp	NE	6	Chinchón (M)	VK6247	640	6-Feb-03	G
*Thymelaea tinctoria* (Pourr.) Endl. subsp. *tinctoria*				Villamayor (Z)			19-Feb-10	
*Thymus lacaitae* Pau	Tl	NE	5	Alcalá de Henares (M)	VK8282	710	28-Jan-03	G

a NE  =  Narrow endemism, G  =  gypsophile, GV  =  gypsovag.

b N  =  number of sampled individuals, C  =  composite sample.

c M  =  Madrid (Central Spain, Middle Tajo Basin), Cu  =  Cuenca (Central Spain, Middle Tajo Basin), Z  =  Zaragoza (NE Spain, Middle Ebro Basin).

Plant species were collected from the two more massive and distinctive gypsum outcrops of the Iberian Peninsula: Central Spain (Middle Tajo Basin, near Madrid) and NE Spain (Middle Ebro Basin, near Zaragoza). Samples were not replicated across both areas owing to the absence of qualitative differences in the spectra of samples of *Ononis tridentata* from different sites (Fig. S1) and the previously reported similar elemental composition of samples collected from both areas [16]. Study species and sampling sites are shown in Table 1, while climatic and edaphic features of each sampling site are in Table 2. All permits required for plant collection at Madrid Community were requested by AE. Permit for sampling of plant material in Aragón was issued by the Government of Aragón (Diputación General de Aragón, DGA) to GMM and SP.

**Table 2:** Climatic and edaphic characteristics of study sites.

Study sites	Area^a^	Climate		Soil				
		T (°C)	P (mm)	pH	SOM (%)	N (%)	CaCO_3_ (%)	Gypsum (%)
Villamayor (Z)	Z	14.2	370	7.7	1.9	0.14	20.0	55.5
Peñaflor (Z)	Z	14.2	370	8.00	0.88	0.051	13.04	66.30
Alcalá de Henares (M)	M	13.2	350	7.8	1.8	0.15	20.6	32.0
Chinchón (M)	M	13.8	422	7.7	2.1	0.12	9.7	59.3
Tendilla (Cu)	M	13.0	410	7.8	2.4	0.17	22.3	29.3

*Abbreviations:* T  =  Mean annual temperature; P  =  total annual rainfall; SOM  =  soil organic matter; N  =  nitrogen.

a M  =  Madrid (Central Spain, Middle Tajo Basin), Z  =  Zaragoza (NE Spain, Middle Ebro Basin).

### Plant and soil sampling and processing

To avoid variability in the chemical composition of plants due to phenology, all plant material was collected during winter, when chemical concentrations of nutrients in the leaves of study plants are steady [16]. Five adult individuals were harvested in each studied population (Table 1). Once in the laboratory, a 15 g sample of the leaves of the short branches of each individual was collected and oven-dried to a constant weight at 60°C. Dry and damaged leaves were excluded from the analyses.

### Chemical analyses

Samples were ground in a ball mill (Retsch Mixer MM301, Leeds, UK) to a fine powder. N and C concentrations were analyzed with an elemental analyzer (Elementar VarioMax N/CN, Hanau, Germany). Subsamples were burnt at 550°C for 4 hours and ash was dissolved in HNO3-HCl-H20 (1∶3∶9) and filtered. Concentrations of Na and K were measured in the soluble (silica-free) ash by flame photometry, Ca and Mg concentrations were determined by complexometry [33] and P concentration was assessed by vanado-molybdate colorimetry [34]. Total sulphur was analyzed by a turbidimetric method with barium chloride [33].

### FTIR Spectroscopic Analysis

As for the other chemical analyses, dried samples for FTIR analysis were prepared by grinding in a ball mill (Retsch Mixer MM301, Leeds, UK) to a fine powder. FTIR spectra were recorded on a Bruker Vertex 70 FTIR spectrometer (Bruker, Ettlingen, Germany) fitted with a potassium bromide beam splitter and a deutroglycine sulphate detector from three replicates of each species and, where the replicates did not match closely, spectra were recorded for two further replicates. A Diamond Attenuated Total Reflectance (DATR) sampling accessory, with a single reflectance system, was used to produce “transmission-like” spectra. Samples were placed directly on a DATR/KRS-5 crystal, and a flat tip powder press was used to achieve even distribution and contact. Spectra were acquired by averaging 200 scans at 4 cm^−1^ resolution over the range 4000 – 370 cm^−1^. A correction was made to the ATR spectra to allow for differences in depth of beam penetration at different wavelengths, using OPUS software (Bruker, Ettlingen, Germany, version 6.0). The spectra were also baseline corrected. No correction was required for water vapour and CO_2_ as the spectrometer is continuously flushed with dry air.

### Statistical analyses

#### Predictions of chemical properties from the FTIR spectra

The relationship between FTIR spectra and the results of the chemical analyses was evaluated through Partial Least Squares (PLS) regression, using the OPUS QUANT version 6.0 software (Bruker, Ettlingen, Germany). Data were available for C (%), N (mg/g), C/N, Na (mg/g), K (mg/g), Ca (%), Mg (mg/g), P (mg/g), S (%) and Ash (%) (Table S1). The validation was done by cross validation, which allows the maximum representation of the samples, essential for such a small dataset. Correlation analyses between “predicted” and “true” values were used to assess the relationships between spectral information and elemental composition of plant samples (R^2^, RMSECV (Root Mean Square Error of Cross Validation), bias and RPD (SD/SECV), Regression Point Displacement, which equals the standard deviation in the field data values divided by the standard error in the Cross Validation error values). There was insufficient representation of all types of species to enable robust calibrations to be developed for prediction of unknown samples, but the data should give a good indication of the extent to which these parameters are related to the IR spectral features.

#### Neural network analysis

The ability of FTIR spectra to discriminate groups of plants with different specificity to gypsum was evaluated by neural network (NN) analyses. Neural networks are particularly useful when the relationships between the input data (in this case the FTIR spectra) and the output parameters (plant species classification) are complex and/or unknown (e.g. [35]). They are also useful when applied to datasets with a large number of input parameters, such as infrared spectra [36]. In order to train the neural network model, the FTIR spectral data was pre-processed to make it more acceptable for NN training. This was carried out in two stages. Firstly, a moving window averaging process was applied to each spectrum, with the moving average at each point subtracted from the actual value. This had the effect of ‘flattening’ the spectra and setting the value of flatter sections of the curve to zero (Fig. S2). The moving window width was set at 101 (averaged over a window that extended 50 values to the left and 50 to the right from each value), following trial and error to optimise the performance of the neural network model. Secondly, the maximum absolute value within each derived spectrum was determined, and the value at each point was divided by this. This had the effect of normalising the spectra within the range [−1, 1] (Fig. S2).

The neural network architecture had four layers, with one input layer of 1882 nodes, two hidden layers each of 100 nodes and one output layer of 3 nodes. The neural network was fully connected (i.e. each node in the input layer was connected to each node in the first hidden layer, and so on). The training rate of the network was set at 0.05, and a total of 10,000 training epochs were used to train the network. These parameter values were selected using trial and error to optimise the model performance. The training algorithm used was the feed-forward back-propagation, a standard method that has been applied in many cases. For further details on this algorithm as applied to environmental data, see [37].

The training data consisted of 45 spectra, which is a relatively small number of data points for training a neural network. To ensure that overfitting of the data was not an issue, a separate neural network was trained for each of the data points, using all of the data except that point and using the one left out for testing purposes. This ‘leave-one-out’ approach ensures that training and testing data do not overlap, and provides more accurate information about how well the model is performing under conditions of low training data numbers. For each data point, the network was trained to predict a value of 1 for output node corresponding to the plant type being presented, and a value of 0 for the other output nodes.

## Results

### Description of FTIR spectra of the different groups

Although the spectra of each species were different and were interpreted individually, this provided a large amount of potentially useful information. Therefore, the results have been summarised for each of the three groups of species analyzed, i.e. “wide gypsophiles”, “narrow gypsum endemisms” and “gypsovags”, and in Table 3 the frequencies and assignments of the main peaks identified are presented.

**Table 3:** Frequencies and assignments of the main peaks identified in the analysis of FTIR spectra.

Wave number, cm^−1^	Assignment	Characterisation
3522,3400	O-H stretching	Gypsum^1^ ^, 2^
3340	O-H stretching	Cellulose, in samples with defined 3340 peak^3^
2920	antisymmetric CH_2_ stretching	Fats, wax, lipids^3^
2850	symmetric CH_2_ stretching	Fats, wax, lipids^3^
1740-1720	C = O stretch of COOR	Esters^3, 4^
1707-1710	C = O stretch of COOH	Carboxylic acids^3, 4^
1653	C = O of amide I	Proteinaceous origin^3^
1615	C-O stretching	Calcium oxalate^5^
1600-1650 (1610)	Aromatic C = C stretching and/or asymmetric C-O stretch in COO-	Lignin and other aromatics, or aromatic or aliphatic carboxylates^3^
1550	N-H in plane (amide-II)	Proteinaceous origin^3^
1505-1515	Aromatic C = C stretching	Lignin/Phenolic backbone^3, 4^
1450-1410	C-O stretching	Calcium carbonate^4, 6^
1426	Symmetric C-O stretch from COO- or stretch and OH deformation (COOH)	Carboxylate/Carboxylic structures (humic acids)^3^
1371, 1450	C-H deformations	Phenolic (lignin) and aliphatic structures^3^
1312	C-O stretching	Calcium oxalate^5^
1265 (approx.)	C-O stretching of phenolic OH and/or arylmethylethers	Indicative of lignin backbone^3^
1265 -1240	C-O-C stretching	Esters
	C-N stretching	Amide III^4^
1140-1080	S-O stretching	Sulphates^4^
1100-1000	P-O stretching	Phosphates^6^
1100-950	Si-O stretching	Silicates^4, 6^
1050 (1030-1080)	Combination of C-O stretching and O-H deformation	Polysaccharides^3^
874	C-O in plane bending	Calcium carbonate^4, 6^
835	Aromatic CH out of plane	Lignin^3^
780	COO bending	Calcium oxalate^5^
720	CH_2_ wag	Long chain (> C4) alkanes^3^
715	C-O in plane bending	Calcium carbonate^4, 6^
680-610	S-O bending	Sulphates^4^
669,597	S-O bending	Gypsum^1^ ^, 2^

1 [39]; ^2^ [56]; ^3^ [38]; ^4^ [21]; ^5^ [57]; ^6^ [58].

**Figure 1:**
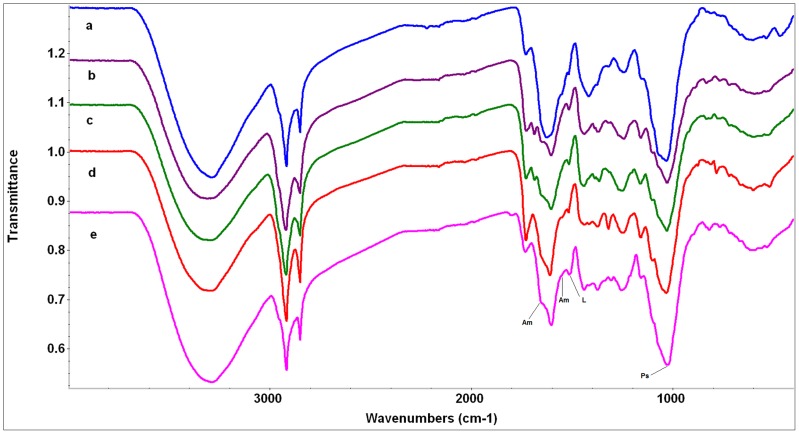
FTIR spectra of gypsovag species. The scale of the Y axis is offset for illustration purposes. a) *Linum suffruticosum* (blue), b) *Rosmarinus officinalis* (purple), c) *Salvia lavandulifolia* (green), d) *Teucrium polium* subsp. *capitatum* (red), e) *Thymelaea tinctoria* (pink). Abbreviations of compound peaks: Am: Amide, L: Lignin/Phenolic backbone, Ps: Polysaccharides.

**Figure 2:**
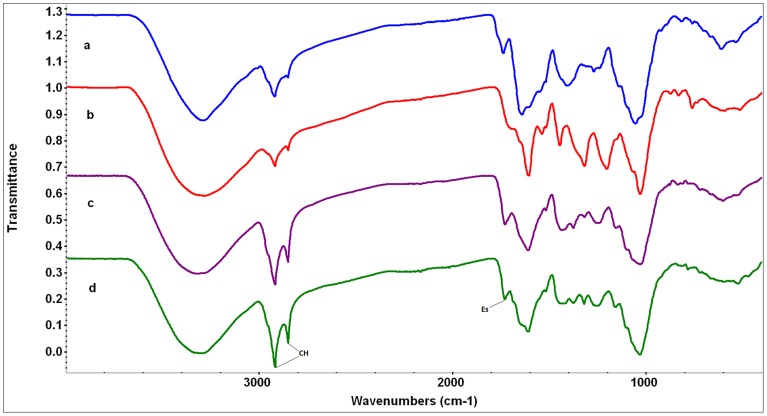
FTIR spectra of narrow gypsum endemisms. The scale of the Y axis is offset for illustration purposes. a) *Centarurea hyssopifolia* (blue), b) *Helianthemum marifolium* subsp. *conquense* (red), c) *Teucrium pumilum* (purple), d) *Thymus lacaitae* (green). Abbreviations of compound peaks: CH: CH-stretching, Es: Esters.

**Figure 3:**
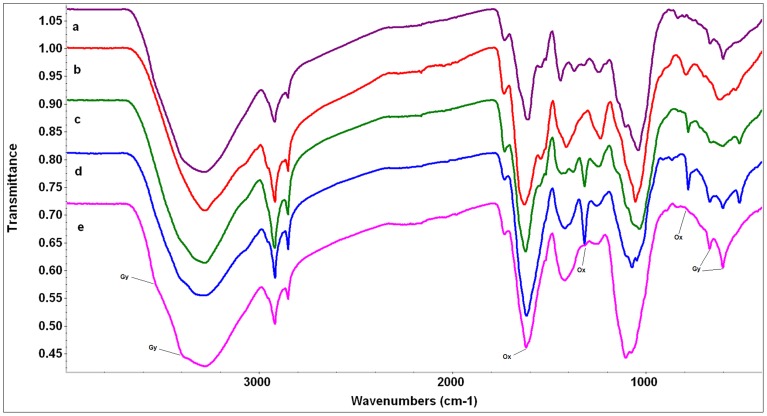
FTIR spectra of wide gypsophiles. The scale of the Y axis is offset for illustration purposes. a) *Helianthemum squamatum* (purple), b) *Lepidium subulatum* (red), c) *Herniaria fructicosa* (green), d) *Gypsophyla struthium* subsp. *hispanica* (blue), e) *Ononis tridentata* (pink). Abbreviations of compound peaks: Gy: Gypsum, Ox: Oxalate.

Overall, the major difference between the spectra of wide gypsophiles and the other two groups is the presence of appreciable gypsum in their spectra (with one exception - see below) and often considerable oxalate too (Figs. 1, 2, and 3, Table 3). In general, the spectra of gypsovags had a lowest proportion of inorganic compounds present in them, while the spectra of narrow gypsum endemisms had a higher proportion, and mostly show the presence of oxalate (Figs. 1, 2, and 3, Table 3). However, for a number of species there was very little difference between the gypsovag and narrow gypsum endemism categories (see below for details).

#### Gypsovags

Replication of the FTIR spectra obtained from gypsovags was generally good (data not shown) with subtle variations in intensities of bands, indicating similar overall chemical profiles within each species. They all exhibited sharp CH_2_ stretching bands, (indicating the presence of long carbon chains) and the same basic spectral patterns with features relating to protein (amide), polysaccharide (cellulose, pectin), lignin, lipids and other long chain hydrocarbons, as might be expected in plant spectra (Fig. 1, Table 3). A feature of the spectra of *R. officinalis* and *S. lavandulifolia* was a distinct band at 1686 cm^−1^, which is possibly an aromatic ester (Fig. 1, Table 3). Notably, in comparison with the spectra of the species in the other two groups, the spectra of gypsovags had the least bands attributable to inorganic components (although all species may have traces). Among the few exceptions were the evidence for calcite, and possibly ammonium sulphate, in the spectrum of *L. suffruticosum*, and oxalate in the spectrum of *T. polium* subsp. *capitatum* (Fig. 1, Table 3).

#### Narrow gypsum endemisms

Replication of the FTIR spectra of narrow gypsophiles was also good, again implying consistent chemical composition within each species. The spectra of this group of species generally appeared to have greater evidence for the presence of inorganic compounds than those of the gypsovag group, although the spectra still showed predominantly features expected from plant material (Fig. 2, Table 3). Apart from *C. hyssopifolia*, where it was not readily detected, the spectra of narrowly distributed gypsophile species all appeared to show the presence of some oxalate. Despite this common feature, there was variation among narrow gypsum endemism in the intensity of the CH_2_ stretching bands in their spectra (indicative of long carbon chains and sometimes referred to as a “waxy” character, [38]). Two of the species (*C. hyssopifolia* and *H. marifolium* subsp. *conquense*) had much weaker CH_2_ absorption in their spectra compared with the spectra of the other two (*T. pumilum* and *T. lacaitae*) which appeared to have stronger absorption, much more similar to the spectra of species in the gypsovag group (Figs. 1–2, Table 3). In fact, *T. pumilum* and *T. lacaitae* had spectra generally quite closely related to those of gypsovags; in particular the spectrum of *T. pumilum* quite closely resembled that of its congener *T. polium* subsp. *capitatum* (including the presence of oxalate). The spectrum of *T. lacaitae* also resembled that of *T. polium* subsp. *capitatum*, and it had also evidence for a band at 1686 cm^−1^(sh), compatible with an aromatic ester, as indicated above for the spectra of other Lamiaceae (*R. officinalis* and *S. lavandulifolia*) of the gypsovag group (Figs. 1–2, Table 3). *C. hyssopifolia* had a spectrum more reminiscent of that of *L. suffruticosum* than the spectra of the other narrow gypsophile species (with a high proportion of protein/amide bands and other bands present which could be assigned to ammonium sulphate or carboxylate). Finally, *H. marifolium* subsp. *conquense* had a spectrum that generally differed from the other narrow gypsophiles, and spectral searches suggested that the presence of tannic acid is a possibility. However it had the oxalate present and relatively strong protein/amide bands, as seemed to be common in the rest of plants from this group (Figs. 1–2, Table 3).

#### Wide gypsophiles

The replication of spectra within this group was noticeably less tight, with variations in intensity of bands, particularly those arising from inorganic components, rather than different bands being present (i.e. same chemistry but different proportions). A distinct feature of the spectra of this group of plants was the relatively high dominance of bands related to inorganic compounds (Fig. 3, Table 3), with clear bands for gypsum in all but one of them (*L. subulatum*). The presence of crystalline gypsum could be confirmed, even in the presence of other sulphates, by shoulders on the broad OH stretch at 3518 and 3398 cm^−1^ (Fig. 3, Table 3). These bands were similar to those detected in the spectra of soil samples collected at the sampling site (data not shown) and had not been identifiable in any of the previous spectra of the gypsovag and narrow gypsophile groups. Oxalate was also strongly present in the spectra of two of the wide gypsophiles analyzed (*H. fruticosa* and *G. struthium* subsp. *hispanica*) and some small traces were also visible in *H. squamatum* and *O. tridentata*, with the most diagnostic band being at 1315 cm^−1^ (Fig. 3, Table 3). Although *L. subulatum*, had a spectrum where inorganic compounds were not so immediately obvious, a broad absorption band in its spectrum at ∼1600 cm^−1^ could possibly be ammonium sulphate or carboxylate, and sulphates other than gypsum may also be present (Fig. 3, Table 3). This spectrum also had the strongest bands relating to amide/protein.

Bands expected from plant material (such as cellulose, lignin, pectin, protein etc) were also present in spectra of wide gypsophiles, but were often obscured by the inorganic compounds (Fig. 3, Table 3). Absorption in the region between 1200 -1000 cm^−1^ could arise from a range of inorganic compounds rather than polysaccharide but this region is very hard to interpret as sulphate, phosphate, polysaccharide and silicate groups all absorb in this frequency range and often have overlapping bands [21], [38], [39].

### Correlation between FTIR spectral data and chemical analyses

In general, FTIR data adjusted reasonably well (very well for some cases) to the concentration of most nutrients in plants (Table 4). Correlation coefficients were above 90.0 for ashes, carbon and sulphur, and above 79.0 for Mg, P, Ca, Na and N. The different forms of N in different species, particularly those in the wide gypsophile group, may result in poorer correlation overall than might be the case if they are separated out, but there were not enough cases to represent each type of N form separately and not enough replicates to separate out each group (Table S1). The main exception was K, which implies that there was not such a clear cut relationship between spectral features and the variance in values of K for the samples analysed.

**Table 4:** Results of PLS regression analyses between FTIR spectra and the C (%), N (mg/g), C/N, Na (mg/g), K (mg/g), Ca (%), Mg (mg/g), P (mg/g), S (%) and Ash (%) concentrations of the samples analysed.

Variable	R^2^	RPD values (Std Dev/Std error)
**C (%)**	95.7	4.81
**N (mg/g)**	79.8	2.24
**Na (mg/g)**	86.2	2.23
**K (mg/g)**	65.1	1.69
**Ca (%)**	88.1	2.69
**Mg (mg/g)**	79.9	2.9
**P (mg/g)**	85.5	2.62
**S (%)**	91.9	3.51
**Ash (%)**	94.5	4.28

### Suitability of FTIR spectra to discriminate ecological strategies of gypsum plants

The neural network (NN) approach was successful in correctly assigning the majority (39 out of 44) of the cases analyzed (Table 5). Predictive accuracies were assessed in terms of the error between target and actual values at each output node (RMSE), and are given in Table 5. RMSE values for gypsovag and narrow endemisms were high. However, they were not as high as would be obtained through purely random output values, for which RMSE values greater than 0.4 would be expected. The RMSE value for gypsophiles was much lower. In general, the NN models built were able to discriminate between the three groups of plants (Table 5). The wide gypsophile category was most accurately predicted with 15 data points correctly predicted out of 15 points analysed. It was followed by gypsovags, with 15 data points correctly predicted out of 17, and narrow gypsum endemisms or narrow gypsophiles, with 9 data points correctly predicted out of 12, and therefore the least accurately predicted group.

**Table 5:** Confusion matrix of plant types, based on neural network model prediction.

Predicted groups	Observed groups		
	Gypsovag	Narrow endemism	Gypsophile
Gypsovag	15	2	0
Narrow endemism	3	9	0
Gypsophile	0	0	15
Model fit			
MAE	0.211	0.196	0.068
RMSE	0.305	0.287	0.098

Values give the number of each type given in the left-hand column that are categorised as each type given in the top row. Bottom rows indicate mean absolute error (MAE) and RMSE (Root Mean Squared Error) values for each output node in the neural network models, in relation to the output errors (range [0, 1]).

Of the five data points that were incorrectly identified, none were from the wide gypsophile category and no other species from either of the other two categories was incorrectly classified as a wide gypsophile. There was, however, some crossover between the spectra of gypsovags and narrow gypsophiles, with five cases being assigned to the wrong group, although generally they could be accurately distinguished. The cases of mis-classification mostly appear to relate directly to the spectral features noted above, i.e. the strong similarity, including the presence of oxalate, between the spectra of *Teucrium polium* subsp. *capitatum* (a gypsovag misclassified as narrow gypsophile) and those of *Teucrium pumilum* and *Thymus lacaitae* (misclassified as gypsovags).

**Figure 4:**
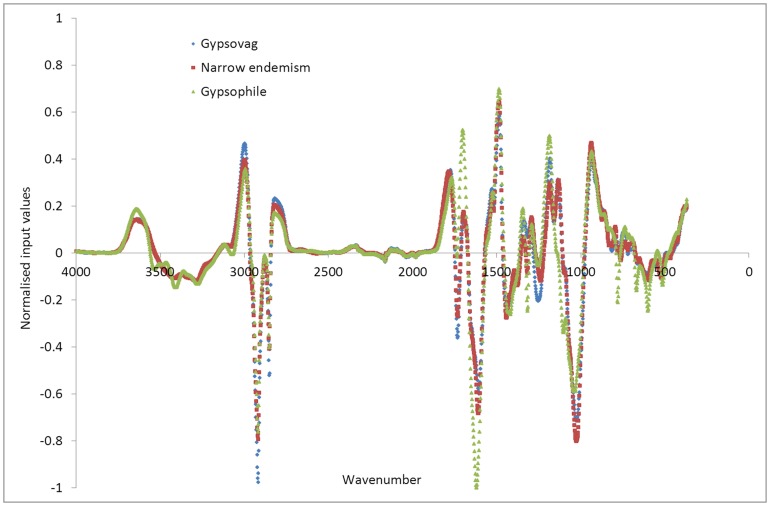
Input datasets averaged over each of the gypsum plant types (gypsovags in blue, narrow gypsum endemisms in red, wide gypsophiles in green), for the full wavenumber range.

**Figure 5:**
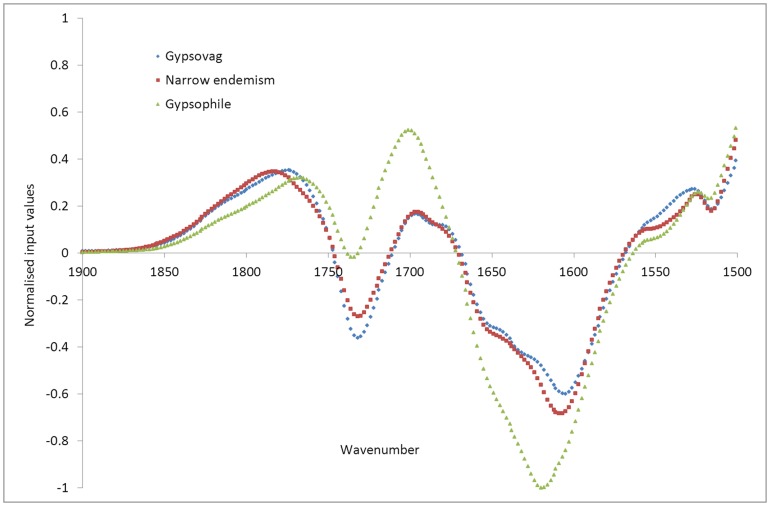
Input datasets averaged over each of the plant types (gypsovags in blue, narrow gypsum endemisms in red, wide gypsophiles in green), for the input wavenumber range 1500 to 1900 cm^−1^.

Visual examination of the input datasets averaged over each group of species (Fig. 4) does not allow much distinction to be drawn between the three types of spectra, although it can be seen that between the frequencies of 3200 and 3600 cm^−1^, and between 1500 and 1900 cm^−1^ (Fig. 5), wide gypsophiles do demonstrate differences to narrow endemisms and gypsovags, which have more subtle differences to each other in these ranges. These ranges, as described above for the FTIR results (Table 3), correspond to bands relating to gypsum present in wide gypsophiles, but not detectable in the other two groups. Also there is variation in oxalate and ester concentrations, with generally lower oxalate in the gypsovags, but higher ester (Fig. 4, Table 3).

**Figure 6:**
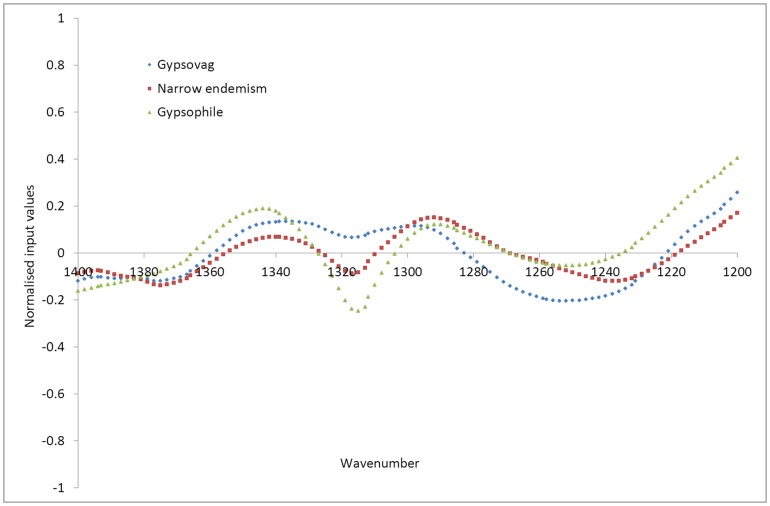
Input datasets averaged over each of the plant types (gypsovags in blue, narrow gypsum endemisms in red, wide gypsophiles in green), for the input wavenumber range 1200 to 1400 cm^−1^.

Examination of the averaged input datasets over smaller ranges however, shows that, while at some ranges narrow gypsum endemisms show similar trends to gypsovags (Figs. 5 and 6), with higher ester (1740 cm^−1^) than wide gypsophiles and no gypsum (1620 cm^−1^), at other ranges narrow gypsum endemisms input value trends are more similar to wide gypsophiles with noticeable (although lower) presence of oxalate (1315 cm^−1^ and 1606 cm^−1^), absent in most gypsovags (Figs. 5, 6). Therefore, it appears that narrow gypsum endemisms, in terms of input values as developed using this FTIR-based approach, show some features both of gypsovags and gypsophiles and can thus be successfully discriminated from them.

## Discussion

The FTIR spectra of the three groups of species analyzed (wide gypsophiles, narrow gypsum endemisms and gypsovags) showed distinct features that enabled their discrimination through neural network (NN) models with relatively high accuracy. This indicates that, although as predicted, elemental and spectral data correlated reasonably well, the analysis of FTIR spectra with NN provided an increased discriminating ability.

As expected, wide gypsophiles stood out for the strong presence of inorganic compounds in their leaves, particularly gypsum. This is in agreement with previous studies on the elemental composition of gypsum plants [12], [13], [14], [16], finding high total ash, Ca and S (mostly in the form of sulphate) concentrations. Some of these studies suggested most of such S could be accumulated in the form of calcium sulphate [13], [14], however, the widespread presence of mineral gypsum (i.e. CaSO_4_•2H_2_O) in gypsophiles has never been reported before. With the sole exception of *L. subulatum*, all the wide gypsophiles analyzed accumulated mineral gypsum in their leaves in the form of crystals. Although previous studies had reported the presence of crystals in some wide gypsophiles like *H. fruticosa* or *G. struthium* subsp. *hispanica* [40], [41], this is the first evidence that most wide gypsophiles studied show such a distinct biochemical trait. Furthermore, the composition of crystals had only been analysed in gypsophiles growing on saline gypsum soils, like *Limonium* spp. or *Frankenia thymifolia* [6], [42]. This is, therefore, the first empirical confirmation that some of the crystals observed in gypsophiles from non-saline soils, such as those considered in this study, are made of gypsum. Wide gypsophiles are commonly considered as “accumulators” of compounds such as S, Ca or Mg, being highly permeable to them and hence able to tolerate high concentrations (toxic for most plant species) in their leaves [12], [13], [14], [43]. Soil gypsum contents above 20–30% are generally considered to be toxic for most plant species [44], but gypsum contents often surpass 50% in the soils where these plants were collected (Table 2). Similarly, normal average concentrations of S in plant leaves are ≈ 0.2% [45], but concentrations between 3 and 6% are common in wide gypsophiles [13], [14], [16], [46]. The ability to “sequester” such excess S in the form of gypsum crystals that accumulate harmlessly in plant cells could be an adaptive mechanism of gypsum-specialist plants. Although gypsum crystals are rare among vascular plants [47], a recent study on the Australian endemic *Acacia robeorum* growing on high S soils, showed that this species was able to accumulate calcium sulphate crystals, presumably as a way to remove excess Ca and S [48]. The ability to accumulate excess gypsum in crystals may be widespread among different taxonomic groups, since the four species that showed gypsum crystals in their leaves in this study belong to three different taxonomic families (i.e. Cariophyllaceae, Fabaceae and Cistaceae). This ability is similar to the common mechanism of halophytes to excrete salts. Although it has been suggested that both mechanisms could be related [41], the question remains if lineages of halophytes and gypsophiles excreting salts are interconnected (i.e. share a common origin), or if both strategies have evolved independently. The general finding of gypsum in the leaves of wide gypsophiles opens up interesting questions regarding the regulatory mechanisms and the processes involved in the metabolism of gypsum in these plants, which is mostly unexplored.

Nevertheless, the formation of gypsum crystals is not the only strategy of wide gypsophiles to cope with high gypsum contents in the soil. *L. subulatum*, a gypsophile common to disturbed gypsum soils of the Iberian Peninsula [31], [49], shows high S concentrations [13], [16], [46], but notably no gypsum. According to our results, this species seems to accumulate S in the form of other sulphates, possibly in combination with N. *L. subulatum* is known for its remarkably high N, amino acid and protein content [16], [46], [50], and high amide and protein bands were found for this species. It is hence possible that part of the S of this species is accumulated as ammonium sulphate (which presence could not be ruled out) or else in the form of S-rich proteins, peptides (glutathione), amino acids (methionine, cysteine) or other organic compounds rich in both N and S such as secondary metabolites (e.g. glucosinolates), which are common among the Brassicaceae. Indeed, the ability of *L. subulatum* to incorporate S into organic compounds has been suggested as a metabolic process explaining *L. subulatum* adaptation to gypsum soils [46].

The spectra of wide gypsophiles were also differentiated by their high calcium oxalate bands, a trait they shared with narrow gypsum endemisms. Calcium oxalate deposition is a common phenomenon in plants, being present in more than 215 plant families [51]. The formation of calcium oxalate crystals in plant tissues is related to various important plant functions, among which the regulation of calcium concentrations seems primary [52]. Accordingly, several studies using a variety of plants have shown that the size and number of Ca oxalate crystals are responsive to changes in the soil concentrations of Ca ([52] and references therein). Previous studies indicated that gypsum specialists are Ca-accumulators [12], [16], [43], a trait that was interpreted as suggestive of the accumulation of mineral gypsum in this type of plants [14]. However, our results indicate that the accumulation of Ca in wide gypsophiles cannot solely be explained by the presence of mineral gypsum. Wide gypsophiles seem to show a further excess of Ca that is accumulated in the form of calcium oxalate. Interestingly, this is a common trait to most narrow gypsum endemisms, which have been suggested to be less specialized to live on gypsum than wide gypsophiles [16]. We therefore suggest that, given the widespread ability to form calcium oxalate crystals in different plants, the liberation of excess Ca in calcium oxalate could be a relatively easy to implement mechanism, as part of the process of adaptation to living on gypsum, of narrow gypsum endemisms.

Iberian gypsovags seem to show physiological mechanisms to block calcium and sulphate uptake by roots. This does not seem to be the case in all gypsovags from other regions of the world. For example, Boukhris and Lossaint [14], [53] found that some species of Tunisian gypsovags were able to accumulate mineral S in their tissues when growing on gypsum, sometimes reaching similar (and even higher) values than gypsophiles. Similarly, Borer et al. [54] identified several strategies to accumulate and excrete excess Ca among gypsovags growing on gypsum soils in the Chihuahuan Desert, possibly involving the formation of calcium oxalate. Although the presence of gypsum was not evaluated by these authors, this discrepancy indicates results from Iberian gypsovags should be extrapolated with care. An increase in glutathione synthesis in leaves has been reported as a signalling factor to regulate sulphate uptake by roots in plants not adapted to gypsum soils [55]. Such a mechanism could also be operating in Iberian gypsovags and gypsum endemisms, but the sulphate metabolism of gypsum plants remains unexplored. The ability of Iberian gypsovags to grow in and out of gypsum soils could be related to their capacity to regulate the uptake of excess nutrients in the soil (particularly S and Ca). Nevertheless, such “blocking” of soil nutrients may also have negative consequences on their ability to uptake other nutrients, such as N and P, which are inherently poor in gypsum soils [18], [19]. This suggestion agrees with the stress tolerant nature of Iberian gypsovags and their reported low nutrient concentrations [16].

Different to previous analyses based only on the elemental composition of gypsum plants [16] where narrow gypsum endemisms were found to be more similar to gypsovags than to wide gypsophiles, our results indicate that local gypsum endemisms share spectral features with the other two groups of gypsum plants that identify them as a separate group. These results have important implications for the understanding of plant adaptation to gypsum substrates as they suggest that narrow gypsum endemisms are not just stress-tolerant plants that find refuge from competition on gypsum without particular specialization to this special substrate. Although, like gypsovags, they do not seem to be able to cope with sulphate accumulation, they share the ability to eliminate excess Ca in calcium oxalate crystals with wide gypsophyles. The narrow gypsum endemisms studied might have recently evolved from stress-tolerant gypsovag species or ecotypes, and could be in the process of specializing to gypsum soils, developing adaptive mechanisms, such as the accumulation of oxalate, to survive on gypsum.

The analysis of FTIR spectra with NN provided an accurate tool to separate the three groups of gypsum plants analyzed. The discrimination ability was decreased for closely related species, such as *T. polium* subsp. *capitatum*, *T. pumilum* or *T. lacaitae*. However, the analysis worked generally well and only 5 out of 44 cases were misclassified. Therefore, this methodology arises as a promising tool for the analysis of plant adaptation to special substrates. Additional future work in this area could focus on determining the chemical composition of plant samples, rather than attempting to discriminate between different functional groups of species. This would provide useful information for nutrition studies and would allow us to determine relationships between soil and plant chemical composition. A NN model that could extract composition information (such as N or S) from FTIR spectra would be more useful than one that provides a categorisation, as it would provide more fundamental information about the samples. To achieve this, it would be necessary to develop calibrations for a wider range of samples and plants from different gypsum areas.

To conclude, FTIR spectroscopy linked to NN analysis is an efficient tool to discriminate plants with different specificity to gypsum substrates. Our results present evidence of the widespread presence of gypsum and calcium oxalate crystals in most gypsum specialist plants studied, although other mechanisms such as the accumulation of sulphates in organic molecules are also compatible with plant specialization to gypsum. While gypsovags seem to be stress tolerant plants that tightly regulate the uptake of S and Ca by their roots, narrow gypsum endemisms share the ability to accumulate excess Ca as oxalate with gypsophiles, possibly indicating their incipient specialization to live on gypsum. Further studies should focus on evaluating the generality of these conclusions and their extrapolation to gypsum plants from other regions of the world.

## Supporting Information

Figure S1 **Comparison of FTIR spectra of** ***Ononis tridentata*** **from different sites: a) red line, Chinchón (Madrid), b) blue line, Villamayor (Zaragoza).**
(TIF)

Figure S2 **Normalisation of FTIR spectra.** Top – raw spectrum, bottom – after moving average preprocessing and normalisation to within range [-1, 1].
(TIF)

Table S1 **Average chemical composition of study species.**
(DOCX)
