# Supplementary figures and images for: Gypsophile Chemistry Unveiled: Fourier Transform Infrared (FTIR) Spectroscopy Provides New Insight into Plant Adaptations to Gypsum Soils
Source: PLoS One. 2014 Sep 15;9(9):e107285. doi: 10.1371/journal.pone.0107285 (PMC4164602; doi:10.1371/journal.pone.0107285)

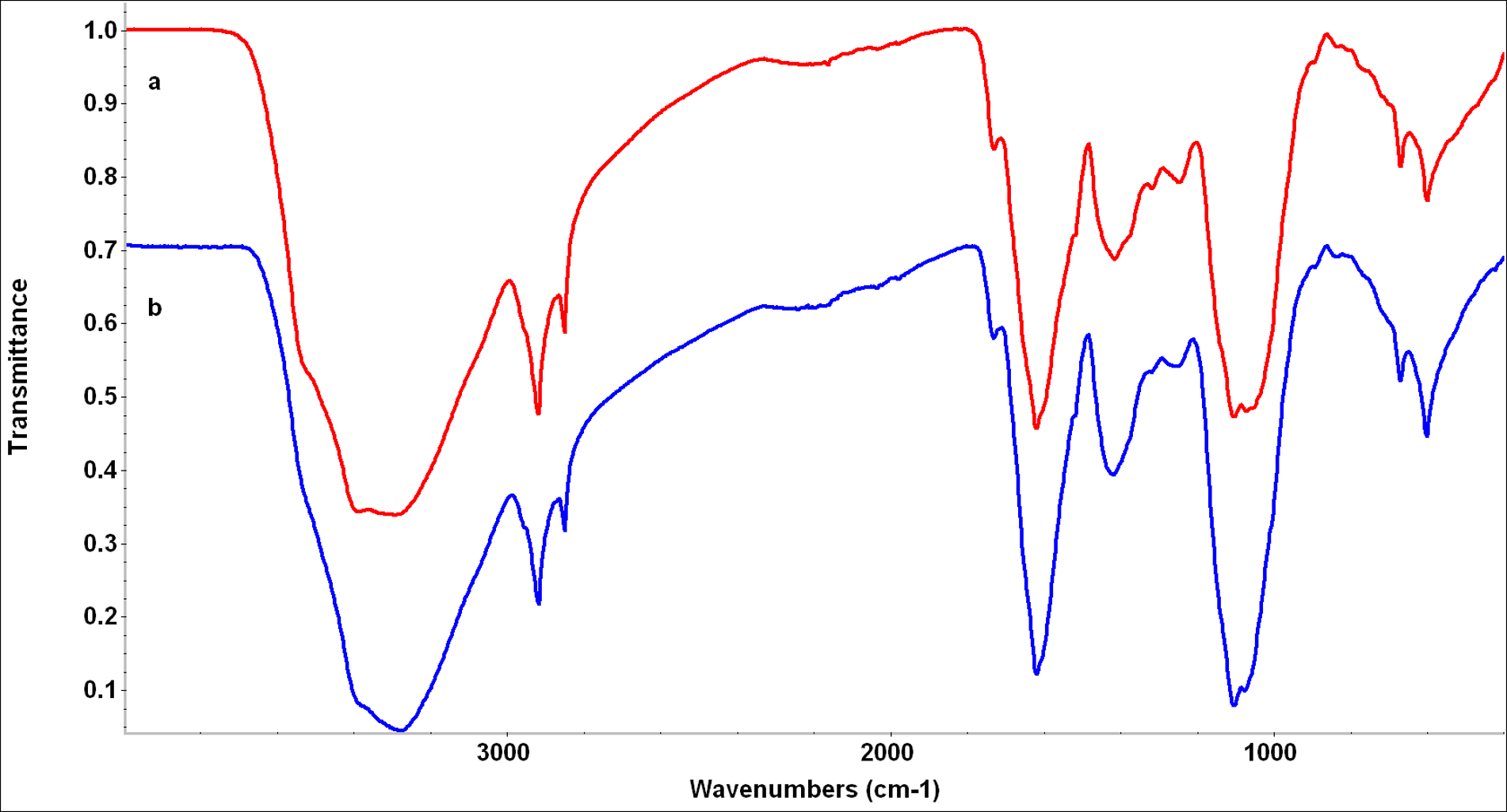

Supplement: Figure S1 — Comparison of FTIR spectra of Ononis tridentata from different sites: a) red line, Chinchón (Madrid), b) blue line, Villamayor (Zaragoza). (TIF) [file pone.0107285.s001.tif]

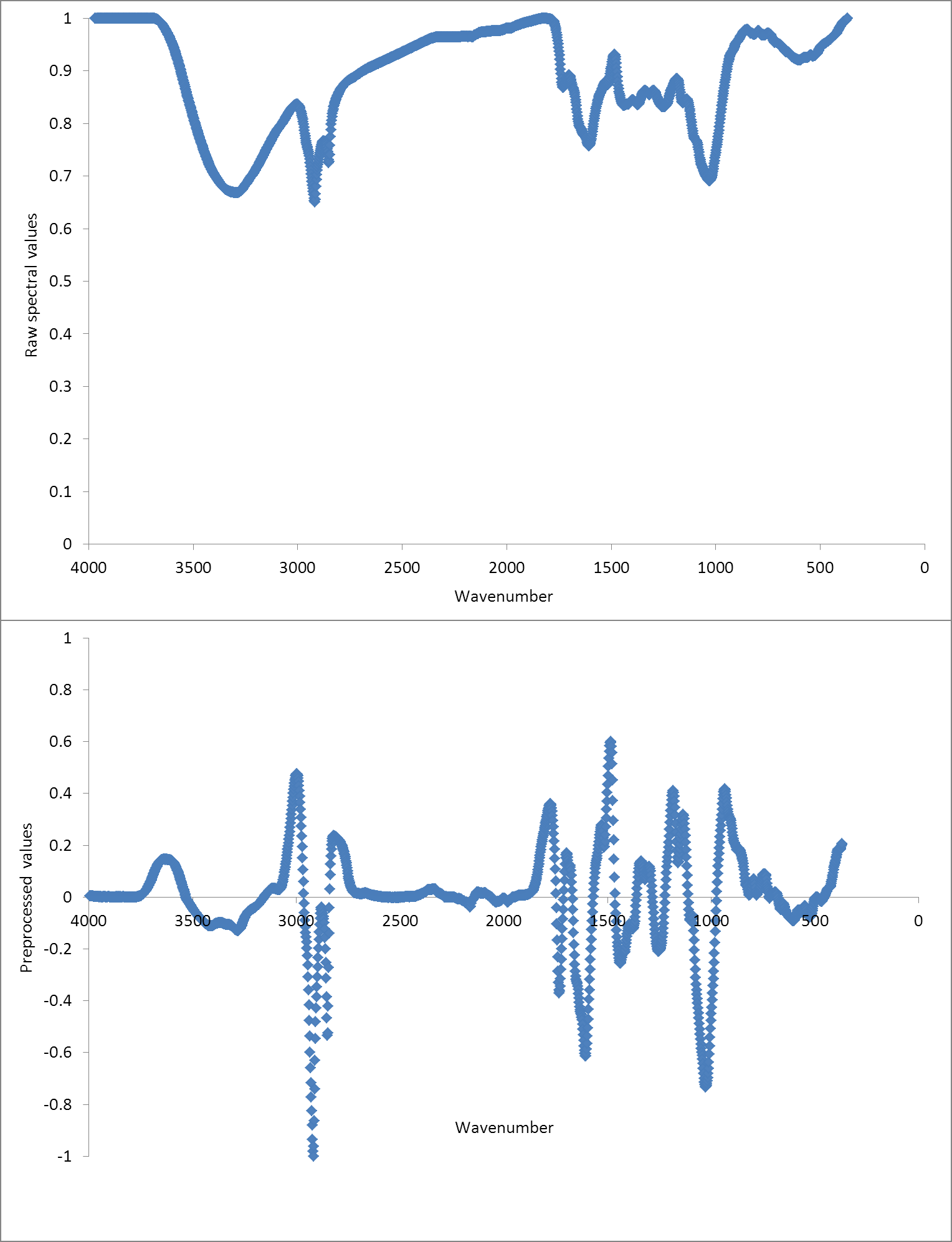

Supplement: Figure S2 — Normalisation of FTIR spectra. Top – raw spectrum, bottom – after moving average preprocessing and normalisation to within range [-1, 1]. (TIF) [file pone.0107285.s002.tif]
