# Supplementary material for: Gypsophile Chemistry Unveiled: Fourier Transform Infrared (FTIR) Spectroscopy Provides New Insight into Plant Adaptations to Gypsum Soils
Source: PLoS One. 2014 Sep 15;9(9):e107285. doi: 10.1371/journal.pone.0107285 (PMC4164602; doi:10.1371/journal.pone.0107285)
Supplement: Table S1 — Average chemical composition of study species. (DOCX) [file pone.0107285.s003.docx]

**Table S1.** Average chemical composition of study species.

| **Species** | **Ash** | **C** | **N** | **P** | **Ca** | **Mg** | **Na** | **K** | **S** |
| --- | --- | --- | --- | --- | --- | --- | --- | --- | --- |
|  | **(%)** | **(%)** | **(mg g ^-1^)** | **(mg g ^-1^)** | **(%)** | **(mg g ^-1^)** | **(mg g ^-1^)** | **(mg g ^-1^)** | **(%)** |
| *C. hyssopifolia* | 14.9 | - | - | 3.1 | 3.5 | 4.4 | 0.6 | 22.0 | 1.5 |
|  | (1.1) |  |  | (0.1) | (0.5) | (1.2) | (0.0) | (3.6) | (0.1) |
| *G. hispanica* | 23.6 | - | - | 1.6 | 6.7 | 12.4 | 0.4 | 9.8 | 3.0 |
|  | (0.7) |  |  | (0.1) | (0.4) | (3.4) | (0.0) | (0.3) | (0.2) |
| *H. conquense* | 6.6 | 49.0 | 15.9 | 0.9 | 1.9 | 2.7 | 0.3 | 3.7 | 0.2 |
|  | (0.1) | (0.1) | (0.1) | (0.0) | (0.0) | (0.3) | (0.1) | (0.2) | (0.0) |
| *H. fruticosa* | 11.6 | 47.4 | 25.3 | 1.0 | 3.0 | 6.4 | 0.5 | 7.7 | 1.0 |
|  | (0.6) | (0.2) | (1.2) | (0.1) | (0.1) | (1.4) | (0.0) | (1.6) | (0.1) |
| *H. squamatum* | 14.1 | 43.7 | 15.8 | 1.1 | 3.4 | 5.5 | 0.7 | 6.3 | 3.0 |
|  | (0.5) | (0.2) | (1.3) | (0.1) | (0.1) | (0.3) | (0.0) | (0.4) | (0.2) |
| *L. subulatum* | 11.5 | 44.9 | 46.6 | 2.7 | 3.2 | 3.3 | 0.5 | 9.8 | 2.5 |
|  | (0.7) | - | - | (0.1) | (0.4) | (0.3) | (0.1) | (0.8) | (0.6) |
| *L. suffruticosum* | 14.6 | 44.0 | 22.5 | 1.4 | 2.7 | 2.6 | 0.6 | 7.0 | 0.1 |
|  | (1.1) | (0.7) | (1.5) | (0.2) | (0.2) | (0.4) | (0.0) | (1.6) | (0.0) |
| *O. tridentata* | 20.9 | 34.6 | 23.6 | 1.0 | 4.4 | 24.8 | 0.5 | 3.1 | 4.0 |
|  | (1.9) | - | - | (0.2) | (0.3) | (2.9) | (0.1) | (0.4) | (0.6) |
| *R. officinalis* | 6.4 | 56.1 | 12.4 | 0.7 | 1.6 | 2.2 | 0.6 | 8.4 | 0.1 |
|  | (0.3) | (0.2) | (0.4) | (0.0) | (0.1) | (0.3) | (0.0) | (0.7) | (0.0) |
| *S. lavandulifolia* | 7.5 | 53.9 | 18.2 | 1.3 | 2.1 | 1.3 | 0.5 | 5.6 | 0.1 |
|  | (0.4) | (0.4) | (0.8) | (0.1) | (0.1) | (0.6) | (0.0) | (0.7) | (0.0) |
| *T. capitatum* | 7.4 | 51.9 | 27.4 | 1.3 | 2.1 | 2.6 | 0.5 | 6.9 | 0.1 |
|  | (0.5) | (0.1) | (2.4) | (0.1) | (0.2) | (0.6) | (0.0) | (1.5) | (0.0) |
| *T. lacaitae* | 8.3 | 51.9 | 14.8 | 1.1 | 1.5 | 3.9 | 0.6 | 6.3 | 0.0 |
|  | (0.2) | (0.2) | (2.2) | (0.1) | (0.1) | (0.5) | (0.0) | (0.4) | (0.0) |
| *T. pumilum* | 8.3 | 52.1 | 16.9 | 0.8 | 2.1 | 6.9 | 0.5 | 7.1 | 0.6 |
|  | (0.3) | (0.1) | (1.8) | (0.2) | (0.0) | (1.0) | (0.0) | (0.6) | (0.2) |
| *T. tinctoria* | 5.9 | 48.9 | 13.3 | 0.9 | 1.5 | 2.8 | 0.1 | 4.9 | 0.1 |
|  | (0.4) | (0.5) | (0.5) | (0.1) | (0.1) | (0.3) | (0.0) | (0.7) | (0.0) |

Data are means (SE in parentheses), N = 3 except for N and C in *L. subulatum* and *O. tridentata* where N = 1.
